# Development and use of real-time PCR to detect and quantify *Mycoplasma haemocanis* and “*Candidatus* Mycoplasma haematoparvum” in dogs

**DOI:** 10.1016/j.vetmic.2009.07.006

**Published:** 2010-01-06

**Authors:** E.N. Barker, S. Tasker, M.J. Day, S.M. Warman, K. Woolley, R. Birtles, K.C. Georges, C.D. Ezeokoli, A. Newaj-Fyzul, M.D. Campbell, O.A.E. Sparagano, S. Cleaveland, C.R. Helps

**Affiliations:** a Department of Clinical Veterinary Science, University of Bristol, Langford House, Langford, Bristol, BS40 5DU, UK; b Disease Ecology Group, Faculty of Veterinary Science, Leahurst Campus, University of Liverpool, Chester High Rd, CH64 7TE, UK; c School of Veterinary Medicine, University of the West Indies, St. Augustine Campus, Trinidad and Tobago; d Newcastle University, School of Agriculture Food and Rural Development, Newcastle upon Tyne, NE1 7RU, UK; e Division of Animal Production and Public Health, Faculty of Veterinary Medicine, University of Glasgow, G61 1QH, UK

**Keywords:** “*Candidatus* Mycoplasma haematoparvum”, *Mycoplasma haemocanis*, Polymerase chain reaction, Prevalence, Ticks

## Abstract

Two canine haemoplasma species have been recognised to date; *Mycoplasma haemocanis* (*Mhc*), which has been associated with anaemia in splenectomised or immunocompromised dogs, and “*Candidatus* Mycoplasma haematoparvum” (*CMhp*), recently described in an anaemic splenectomised dog undergoing chemotherapy. The study aim was to develop quantitative real-time PCR assays (qPCRs) incorporating an endogenous internal control to detect *Mhc* and *CMhp* and to apply these assays to DNA samples extracted from canine blood collected in Northern Tanzania (*n* = 100) and from dogs presented to a Trinidadian veterinary hospital (*n* = 185).

QPCRs specific for *Mhc* and *CMhp* were designed using 16S rRNA gene sequence data, and each was duplexed with an assay specific for canine glyceraldehyde-3-phosphate dehydrogenase (GAPDH). The assays detected ≤10 copies of a sequence-specific haemoplasma plasmid per reaction and neither assay showed cross-reactivity with 10^6^ copies of the sequence-specific plasmid from the non-target canine haemoplasma species.

Nineteen of the 100 Tanzanian samples (19%) were positive for *Mhc* alone and one (1%) was dually infected. One Trinidadian sample was negative for canine GAPDH DNA and was excluded from the study. Of the 184 remaining Trinidadian samples, nine (4.9%) were positive for *Mhc* alone, five (2.7%) for *CMhp* alone, and two (1.1%) dually infected.

This is the first report of canine haemoplasma qPCR assays that use an internal control to confirm the presence of amplifiable sample DNA, and their application to prevalence studies. *Mhc* was the most commonly detected canine haemoplasma species.

## 1 Introduction

Haemoplasmas are epierythrocytic parasites of mammals that are currently uncultivatable *in vitro*. They were reclassified from the *Haemobartonella* and *Eperythrozoon* genera to the *Mycoplasma* genus due to 16S rRNA gene sequence analysis (Messick et al., 2002). Infection with *Mycoplasma haemocanis* (*Mhc*) generally only induces clinically significant anaemia in splenectomised or immunocompromised dogs, although latent infections may cause sub-clinical anaemia (Brinson and Messick, 2001). “*Candidatus* Mycoplasma haematoparvum” (*CMhp*) was first described in association with anaemia in a splenectomised dog undergoing chemotherapy for leukaemia (Sykes et al., 2004).

Recently, quantitative real-time PCR (qPCR) assays have been described for the detection of *Mhc* and *CMhp* 16S rRNA genes in blood samples (Kenny et al., 2004; Wengi et al., 2008). However, none of these qPCR assays include an endogenous internal control to confirm the presence of amplifiable canine DNA and the absence of PCR inhibitors.

Two prevalence studies, from southern France and eastern Sudan, found *CMhp* to be more prevalent than *Mhc*, with occasional co-infections also detected (Kenny et al., 2004; Inokuma et al., 2006). A recent Swiss study reported much lower *CMhp* and *Mhc* prevalences (Wengi et al., 2008). It has been suggested that these differences in haemoplasma prevalence could be due to the absence of the proposed tick vector, *Rhipicephalus sanguineus*, in most of Switzerland compared to its presence in southern France and eastern Sudan (Wengi et al., 2008).

The aim of this study was to develop species-specific *Taq*Man qPCR assays, containing an endogenous internal control, for the diagnosis of canine haemoplasma infections and to use these assays to determine the prevalence of infection in dogs from geographical areas where the proposed vector *R. sanguineus* is widespread (Walker et al., 2000).

## 2 Materials and methods

*CMhp*-specific primers and probe were designed to the 16S rRNA gene at sites of divergence from Mhc and “Candidatus Mycoplasma haemominutum” (*CMhm*) using Primer 3 (Rozen and Skaletsky, 2000). *Mycoplasma haemofelis* (*Mhf*)-specific primers and probe were utilised for the *Mhc* qPCR due to sequence identity (Peters et al., 2008). *Mhc* and *CMhp* qPCR assays were duplexed with a previously validated canine GAPDH gene-specific qPCR as internal control (Peters et al., 2003) (Table 1).

Both duplex qPCRs were optimised for primer/probe concentration and annealing temperature. QPCR was performed using 2× Qiagen HotStarTaq Master Mix (Qiagen, Crawley, UK) with 200 nM haemoplasma primers, 100 nM haemoplasma *Taq*Man probe, 25 nM canine GAPDH primers, 50 nM canine GAPDH *Taq*Man probe, 4.5 mM final MgCl_2_ and 5 μl gDNA in a total volume of 25 μl. All reactions were performed in an iCycler IQ (Bio-Rad Labs Ltd., Hemel Hempstead, UK) at: 95 °C for 15 min and 45 cycles of 95 °C for 10 s and 60 °C (*Mhc*) or 62 °C (*CMhp*) for 30 s, during which fluorescence data were collected.

Due to the near-100% sequence identity between *Mhc* and *Mhf*, a previously sequenced plasmid containing the entire *Mhf* 16S rRNA gene (Tasker et al., 2003) was used to test the efficiency and specificity of the *Mhc* qPCR. A 1458 bp near-complete 16S rRNA gene product was amplified from a *CMhp*-positive sample, using universal prokaryote primers 8F and 1492R (Pitulle et al., 1999). The appropriate sized fragment was gel purified, cloned into pCR4-TOPO (Invitrogen) and sequenced by The Sequencing Service (School of Life Sciences, University of Dundee, Scotland). The sequence was subjected to BLASTn (www.ncbi.nlm.nih.gov) and compared to previously published *CMhp* sequences.

These plasmids were used to determine haemoplasma reaction specificity, sensitivity and efficiency. Assays were conducted in a background of canine gDNA (approximately 70 ng/reaction). Haemoplasma qPCR specificity was tested using 10^6^ copies of the non-target 16S rRNA gene sequence as template. A 10-fold serial dilution of each plasmid, 10^6^ to one copy per qPCR for the *CMhp* assay, and 10^7^ to one copy per qPCR for the *Mhc* assay, were generated to determine assay sensitivity. Plots of threshold cycle (*C*_t_) versus log_10_ copy number were used to determine qPCR efficiency.

Intra-assay and inter-assay precision were measured by analysis of aliquotted DNA samples on the same (*n* = 6 for each plasmid concentration) and separate plates (*n* = 4). Coefficients of variation (CVs) were calculated for copy number and *C*_t_ at high (10^6^ copies of *Mhc*; 10^5^ copies of *CMhp*) and low (10^2^ copies of *Mhc*; 10 copies of *CMhp*) plasmid concentrations. Relative copy numbers were calculated from replicate *C*_t_ values assuming 40 equalled 1 haemoplasma copy/reaction, and using the reaction efficiencies obtained from the standard curves.

Blood samples (*n* = 100) were taken as part of the Carnivore Disease Project's research programme in northern Tanzania during June 2007. Clinical data including sex, age and presence/absence of *R. sanguineus* ticks were recorded. Blood was applied directly to Whatman FTA Classic cards (Whatman Inc., Clifton NJ, USA), air dried and stored at room temperature. Two 2 mm diameter discs were taken from each sample using individual sterile punches (Miltex, Inc., York, USA) and digested in 180 μl T1 Buffer with 20 μl Proteinase K (overnight; 56 °C, 1000 rpm in a Vortemp 56 shaker). DNA extraction was performed using a Macherey-Nagel Nucleospin Blood kit (ABgene, Epson, UK) with DNA eluted into 100 μl of elution buffer. Negative control extractions were performed with each batch.

DNA samples (*n* = 185) were extracted from whole blood obtained from a veterinary diagnostic laboratory in Trinidad between July 2004 and March 2006 as part of another study (Georges et al., 2008), using a QIAamp^®^ DNA blood kit (QIAGEN^®^, Valencia, CA, USA) and stored at −20 °C until use. Age, sex and haematological parameters were recorded.

All DNA samples from Tanzania and Trinidad were subjected to the *Mhc* and *CMhp* qPCR assays. Positive control samples (of known copy number) and negative controls (water) were included on each PCR plate.

Data were entered into Excel (Microsoft^®^ 2002) and analysed or imported into SPSS for Windows (SPSS Inc., Chicago IL, USA). The 95% confidence intervals (CI) of the observed prevalences were calculated. Age and haematological variables were tested for normality using the Kolmogorov–Smirnov test. Normally and non-normally distributed data were analysed using the Student's *t*-test and Mann–Whitney test respectively. Categorical data were analysed using the *χ*^2^-test. Significance was taken as *P* ≤ 0.05.

## 3 Results

BLASTn analysis of the 1458 bp 16S rRNA gene fragment from *CMhp* confirmed it to be closely related to previously published *CMhp* sequences (99.4–99.9% identity). No cross-reactivity was detected for either qPCR when 10^6^ copies/reaction of the non-target species plasmid was used as template. The *Mhc* qPCR had a reaction efficiency of 95.5% (*r*^2^ = 0.994) and consistently detected 10 copies per reaction. At one copy per reaction one of three replicates was detected. The *CMhp* qPCR had a reaction efficiency of 93.9% (*r*^2^ = 0.997) and detected one copy per reaction in all three replicates. For the qPCR standard curves *C*_t_ values ranged from 15.8 to 38.8 for *Mhc* and 14.6 to 40.3 for *CMhp*; in these reactions GAPDH *C*_t_ values ranged from 17.7 to 21.2. The CVs for the detection of *Mhc* and *CMhp* are shown in Table 2.

All 100 Tanzanian samples were positive for canine GAPDH DNA (*C*_t_ values 20.7–27.8). Forty-five percent (*n* = 45) were from male dogs and 55% (*n* = 55) from females, ages ranged from 2 months to 7 years (median 2 years) and, where records were available, 55.2% (*n* = 48) had at least one *R. sanguineus* tick. Nineteen dogs (19%) were positive for *Mhc* alone, and one was co-infected with *Mhc* and *CMhp*. Males were more likely to be *Mhc* positive than females (*χ*^2^, *P* = 0.024), with 31.1% (*n* = 14) males positive compared to 10.9% (*n* = 6) females. No significant association was found between haemoplasma status and age (M-W, *P* = 0.732) or presence of ticks (*χ*^2^, *P* = 0.708).

Of the 185 Trinidadian samples, 184 were positive for canine GAPDH DNA (*C*_t_ values 15.3–27.2) whilst one was negative and thus excluded from the study. Males made up 55.4% (*n* = 102) of the population whilst 44.6% (*n* = 83) were female and ages (*n* = 183) ranged from 2 months to 15 years (median 6.0 years). Red blood cell counts were 5.24 ± 1.5 × 10^12^ cells/l (mean ± SD; reference range: 5.5–8.3 × 10^12^ cells/l); haemoglobin 121.4 ± 35.8 g/l (reference range: 120–180 g/l) and haematocrit 0.346 ± 0.098 l/l (reference range: 0.37–0.55 l/l). Of the 184 GAPDH positive samples 4.9% (*n* = 9) were infected with *Mhc* alone, 2.7% (*n* = 5) with *CMhp* alone and 1.1% (*n* = 2) were dual infected. No significant association was found between haemoplasma status and age (M-W, *P* = 0.896), sex (*χ*^2^, *P* = 0.708) or haematological parameters (*t*-tests; RBC count *P* = 0.175; haemoglobin concentration *P* = 0.597; haematocrit *P* = 0.633).

All of the qPCR water and extraction controls were negative for both GAPDH and haemoplasma. All of the positive controls were positive at the expected *C*_t_ value for both GAPDH and the respective haemoplasma.

## 4 Discussion

This study reports, for the first time, the development of species-specific 16S rRNA gene-based qPCR assays duplexed with a canine GAPDH internal control assay for the diagnosis of canine haemoplasma infection. The inclusion of the internal control is important in order to prevent false negative results due to the failure of DNA extraction, presence of PCR inhibitors or qPCR setup errors. Both haemoplasma qPCR assays described in this study could detect between one and 10 copies per PCR in the presence of canine genomic DNA. Our results were comparable with sensitivities reported for previously published qPCR canine and feline haemoplasma assays (Peters et al., 2008; Wengi et al., 2008).

The newly described qPCR assays were successfully applied to DNA extracted from whole anti-coagulated blood and Whatman FTA Classic cards. Whilst Whatman FTA Classic cards are a solution to atypical environmental conditions, samples had to undergo additional processing steps, compared to whole anti-coagulated blood, in order to extract DNA, which could have resulted in insufficient DNA in the resultant preparation or the presence of PCR inhibitors. Comparable levels of GAPDH DNA were detected in both the Tanzanian and Trinidadian samples. The GAPDH DNA negative Trinidadian sample was also negative for both species of haemoplasma, and could have been mistakenly included in the prevalence calculations without the internal control assay.

Both canine haemoplasma species were detected in the Tanzanian and Trinidadian samples tested in this study. However, in contrast to previous studies in Sudan and France (Kenny et al., 2004; Inokuma et al., 2006), *Mhc* was more prevalent than *CMhp*. Similar populations of representative village dogs were used in both the Sudanese and Tanzanian studies, in contrast to the veterinary clinic derived samples from typically sick dogs used in the French, Swiss (Wengi et al., 2008), and our Trinidadian studies. The French study reported an overall *Mhc* prevalence of 5.9% which was similar to our Trinidadian population (6.0%). However, the French prevalence of *CMhp* (12.2%) was much greater than that in Trinidad (3.8%) although difficulties exist in comparing results since small sample sizes may not necessarily truly represent the whole of the local population.

The French and Sudanese studies were from climactic regions known to support the proposed vector *R. sanguineus*’ life cycle (Kenny et al., 2004; Inokuma et al., 2006). In contrast, the climate across most of Switzerland is too cool to support *R. sanguineus*, and all the haemoplasma positive dogs identified in that study (0.3% *CMhp* and 0.9% *Mhc* positive; no co-infections; *n* = 889) had either originated from, or had visited areas where *R. sanguineus* was known to be prevalent (Wengi et al., 2008). No association was found between tick presence and haemoplasma status in our Tanzanian samples. This was not unexpected since haemoplasmas can chronically infect animals (Wengi et al., 2008) such that a direct temporal relationship between tick and haemoplasma infections may not always occur.

The Swiss study found no relationship between haemoplasma (*Mhc* and *CMhp*) infection and the presence of anaemia (Wengi et al., 2008), similar to our findings for the Trinidadian samples. Both the Swiss and the French studies found no association between age or sex and haemoplasma status (Kenny et al., 2004; Wengi et al., 2008), again similar to our Trinidadian samples; although males were significantly more likely to be *Mhc* positive in the Tanzanian samples. A recent study found that 34.2% of 73 Tosas (Japanese fighting dogs) were *Mhc* positive compared to 1.4% of 840 non-Tosa dogs sampled (Sasaki et al., 2008). Of the Tosa *Mhc* positive dogs, 96% were male. The increased prevalence of *Mhc* in Tosas was attributed to ingestion of infected blood during aggressive contact. The domestic dogs sampled in Europe and Trinidad are not likely to have roaming or fighting tendencies whereas in Tanzania the majority of rural dogs are free-roaming with frequent opportunities for fighting, especially amongst males which may explain the increased haemoplasma prevalence amongst the Tanzanian male dogs.

## 5 Conclusion

We have described the development and use of qPCRs for the detection of canine haemoplasmas, which include an internal control to confirm the presence of amplifiable DNA from canine blood preparations. We also describe the application of these assays to blood derived DNA from two separate populations of dogs, both residing in areas harbouring *R. sanguineus*. Comparable levels of haemoplasma infection to other areas where the tick vector is prevalent were found, albeit with different ratios of infecting species.

## Figures and Tables

**Table 1:** Details of the qPCR assays used for the detection of haemoplasma species (16S rRNA gene) and canine DNA (glyceraldehyde-3-phosphate dehydrogenase gene). *Mhc*: *Mycoplasma haemocanis*; *CMhp*: “*Candidatus* Mycoplasma haematoparvum”. FAM: 6-carboxyfluorescein; TXR: Texas Red; BHQ1/2: Black Hole Quencher^®^ 1/2.

Target	Forward primer (5′–3′)	Reverse primer (5′–3′)	Probe (5′–3′)
*Mhc*	GTGCTACAATGGCGAACACA	TCCTATCCGAACTGAGACGAA	FAM-TGTGTTGCAAACCAGCGATGGT-BHQ1
*CMhp*	GGAGAATAGCAATCCGAAAGG	GCATTTACCCCACCAACAAC	FAM-CTTCGGGAGCCCCGCGC-BHQ1
Canine	TCAACGGATTTGGCCGTATTGG	TGAAGGGGTCATTGATGGCG	TXR-CAGGGCTGCTTTTAACTCTGGCAAAGTGGA-BHQ2

**Table 2:** Coefficients of variation for canine haemoplasma qPCR assays—based on calculated copy number (and threshold cycle). *Mhc*: *Mycoplasma haemocanis*; *CMhp*: “*Candidatus* Mycoplasma haematoparvum”.

Assay	*Mhc*	*CMhp*
Intra-assay—high [plasmid]	9.2% (0.87%)	9.2% (0.79%)
Intra-assay—low [plasmid]	19.8% (0.98%)	17.6% (0.86%)
Inter-assay—high [plasmid]	4.7% (0.43%)	5.1% (0.40%)
Inter-assay—low [plasmid]	2.8% (0.13%)	13.5% (0.66%)
